# Caregiver and Birth Parent Influences on Depression and Anxiety in African American Children in Kinship Care

**DOI:** 10.3390/healthcare13162025

**Published:** 2025-08-17

**Authors:** Tyreasa Washington, Sheryl L. Coley, Joan M. Blakey, Quenette L. Walton, Jeff Labban, Helen B. Tadese, Dominique N. Martinez, Sonya J. Leathers

**Affiliations:** 1 Department of Social Work, University of North Carolina at Greensboro, P.O. Box 26170, Greensboro, NC 27402, USA; 2 Child Trends, 12300 Twinbrook Parkway, Suite 235, Rockville, MD 20852, USA; dmartinez@childtrends.org; 3 Department of Psychiatry, Harvard Medical School, Boston, MA 02115, USA; 4 Department of Public Health Education, School of Health and Human Sciences, University of North Carolina at Greensboro, P.O. Box 26170, Greensboro, NC 27402, USA; slcoley@uncg.edu; 5 School of Social Work, University of Minnesota—Twin Cities, 1404 Gortner Avenue|105 Peters Hall, Saint Paul, MN 55018, USA; blak0014@umn.edu; 6 Graduate College of Social Work, University of Houston, 3511 Cullen Blvd, Room 117, Houston, TX 77204, USA; qwalton2@central.uh.edu; 7 Office of Research, School of Health and Human Sciences, University of North Carolina at Greensboro, P.O. Box 26170, Greensboro, NC 27402, USA; jdlabban@uncg.edu (J.L.); hbtadese@uncg.edu (H.B.T.); 8 CEHHS Office of Research and External Funding, University of Tennessee, 322 HPER Building, 1914 Andy Holt Avenue, Knoxville, TN 37996, USA; 9 College of Health and Human Sciences, School of Social Work, University of North Carolina at Wilmington, Wilmington, NC 28403, USA; 10 Jane Addams College of Social Work, University of Illinois Chicago, 1040 West Harrison Street, Chicago, IL 60607, USA; sonyal@uic.edu

**Keywords:** child welfare, kinship care, black children/black families, depression, anxiety, mental health

## Abstract

**Background/Objectives**: Depression and anxiety in children pose a significant public health concern, with long-term implications for well-being. Over 10% of children and adolescents are affected by emotional disorders such as depression and anxiety. African American youth face disproportionate exposure to mental health risk factors, including poverty, adverse childhood events, community violence, and racial discrimination, which elevate their vulnerability to these disorders. A particularly at-risk subgroup includes African American children in kinship care arrangements (e.g., grandparents raising grandchildren), who may face additional factors such as family disruption and separation from birth parents. **Methods**: This mixed-methods sequential study examined how caregiver stress and birth mother–child relationship quality relate to depression and anxiety symptoms in African American children in kinship care. Phase I included survey data from 58 caregivers of children aged 5 to 12; Phase II involved interviews with 16 of these caregivers. **Results**: Results indicated that lower caregiver stress was associated with reduced child depression and anxiety symptoms. Furthermore, findings suggest that a high quality of the birth mother–child relationship serves as a promotive factor, particularly for depressive symptoms. Qualitative findings highlighted two themes: (1) the weight of kinship care, marked by factors such as ongoing grief and financial strain; and (2) birth parent relationships, defined by a mix of connection, conflict, and loss that affects children’s mental health. **Conclusions**: These findings underscore the need for greater understanding of the strengths and resources within kinship families that support positive mental health outcomes and highlight the importance of targeted interventions to reduce caregiver stress and foster supportive parent–child relationships.

## 1. Introduction

Mental health disorders such as depression and anxiety in children represent a significant public health challenge, and they have implications for their overall well-being. Research trends have indicated that over 10% of children and adolescents have emotional disorders, including depression and anxiety [1,2]. Prior to 2020, approximately one-fifth of adolescents had at least one episode of major depression, and nearly 1 in 10 children and adolescents had diagnoses of anxiety or other mental disorders [3]. Some possible negative effects of depression and anxiety for children are low academic performance, impaired functioning, severe challenges in their personal and family lives, and suicide if left untreated [4,5]. In a recent survey of adolescent mental health from 2020 through mid-2021, 19.9% of adolescents thought about suicide, and attempts occurred among 9% of adolescents [6].

When compared with youth of other racial and ethnic groups, African American youth are often more exposed to negative risk factors affecting mental health, including poverty, adverse childhood events, community violence, and racial discrimination, all of which may increase their risk of depression and anxiety symptoms [7,8]. A subgroup of African American children reflects those in a living arrangement where related persons are their primary caregivers, called kinship care (e.g., grandparents raising grandchildren). African American children who reside in kinship care may have additional risk factors for depression and anxiety, such as the circumstances surrounding reasons for kinship care and the distress of being separated from their birth parents. Given the challenges some African American children face—particularly those in kinship care—research is needed to identify the strengths and resources in kinship families that support positive mental health outcomes. This mixed-methods study aimed to explore the impact of caregivers’ stress and the quality of birth mother–child relationships on child depression and anxiety.

### 1.1. Understanding Kinship Care

Kinship care occurs when children who cannot live with their biological parents are placed in the custodial care of relatives or persons close to the children’s families (e.g., godparent, church member) [9,10]. Arrangements for kinship care are typed as either formal or informal. Formal kinship care is arranged and supervised by child welfare agencies, whereas children in informal kinship care are not supervised by the child welfare system. Kinship care placements have increased in the past few decades; for many child welfare stakeholders, it is the preferred type of out-of-home placement for children as an alternative strategy for traditional foster care [10]. Approximately 2.5 million children live in kinship care arrangements [9,11]. Grandparents consistently comprise the largest proportion of kinship caregivers. Nationally, as of 2020, approximately 2 million grandparents were primary caregivers for their grandchildren [12], and more than 1 million relatives other than grandparents cared for related children during this time frame [13].

### 1.2. African Americans and Kinship Care

There is an overrepresentation of African American children who experience kinship care when compared to children of other racial and ethnic groups [14,15]. Many members of African American communities, researchers, and scholars have attributed the disproportionate number of African Americans engaged in kinship care to social inequities and structural racism. For example, this type of placement became necessary during slavery and the forced splitting of families, when other enslaved extended persons stepped up to take care of children separated from their birth parents [16,17]. Additionally, the need for kinship care among African Americans continued during events such as the Great Migration in the mid-20th century, when children remained in southern states with relatives while their parents searched for economic and educational opportunities in northern and central states [18]. Kinship care has remained a strength and valuable resource for African American families, supporting advancement and adaptation in the face of systemic oppression. Research has found that kinship care is often used as a living arrangement when parents are involved with the criminal justice and child welfare systems, both of which impact the African American community disproportionately [19,20,21,22]. Given the overrepresentation of African American kinship care families, it is imperative to conduct research specific to this population to identify strategies to promote positive outcomes for these children and families.

### 1.3. Depression and Anxiety Outcomes Among Children

Concerns about depression and anxiety among youth have persisted, with recent trends indicating that approximately one in five children and adolescents experience significant mental, behavioral, and emotional distress [5,23] and over 10% suffer from depression, anxiety, and other disorders that impair their personal, school, and family lives [1,2,5]. Depression and anxiety often co-occur as mental health conditions [2,24,25,26], further compounding their negative effects on the academic functioning and personal and family lives of children and adolescents [5].

Research on depression and anxiety among racial and ethnic adolescent groups shows mixed findings [27]. Some studies report higher rates of depressive symptoms and anxiety disorders among non-White youth [28,29,30], while others find equivalent or lower rates of depression among Black adolescents compared to White or Hispanic peers, but higher anxiety and comorbidity [31,32,33,34]. Black youth face disproportionate exposure to risk factors—poverty, violence, discrimination—that elevate their mental health risks [7,8,35]. For instance, more Black youth live below the poverty line, which predicts mental disorders [36,37]. In one study, 41% of Black adolescents reported trauma linked to anxiety and depression [7]. Given these vulnerabilities, it is critical to examine protective factors, particularly in subgroups such as children in kinship care, whose unique circumstances may further shape mental health outcomes.

### 1.4. Outcomes Among Children in Kinship Care

Children in foster care experience higher rates of health issues, behavior problems, depression, anxiety, relational challenges, and academic difficulties compared to the general population [38,39]. However, outcomes vary by placement type. Kinship care has been associated with significant benefits, including stronger family and community connections, cultural continuity, and greater placement stability [9,40,41,42]. Research also indicates that children in kinship care are less likely to experience re-abuse and live in safer, more secure environments [43,44,45].

Academic outcomes are mixed. Washington et al. (2021) [46] found that children in kinship care had higher test scores than those in nonrelative foster care, with scores for those in formal kinship care aligning with general population norms. However, Font (2014) [47] reported declining reading scores over time in a national sample.

Most studies report fewer behavior problems among children initially placed in kinship care, especially among older children and those with fewer initial concerns [47,48,49]. While few studies isolate anxiety and depression, one Canadian study found no significant differences between kinship and nonrelative foster care [50], while another found reduced symptoms in kinship care over time [47].

Despite less emphasis on internalizing symptoms in past research, studies show high rates of anxiety and depression among children in both kinship and traditional care. One nationally representative study found caregiver-reported depression and anxiety in 14.2% of children—more than four times the rate in the general population [39]. Similarly, over 20% of children in out-of-home care had clinically significant internalizing scores [38]. Although some children placed with kin may experience reductions in mental health symptoms over time, their levels of depression and anxiety remain high.

### 1.5. Caregiver Stress

Despite the benefits of kinship care for children and positive reports from their caregivers about their parental roles, kinship caregivers can potentially experience stress at high rates, which could have harmful effects on their well-being. For example, Sharda et al. (2019) [51] found negative effects of parental stress, physical, and psychosocial quality as measured by the Quality of Life survey within a sample of 152 kinship caregivers. Complications appear to impact grandparents raising grandchildren disproportionately, as they potentially have greater parenting stress and worse health than other caregivers [52].

Stressors for caregivers can come from multiple circumstances. According to Gleeson et al. (2016) [53], parenting stress for kinship caregivers can be influenced by social support, family resources, and family competence. Families that report high levels of help from social support, adequate family resources, and positive family functioning capabilities could have lower levels of parenting stress. Kinship caregivers could also experience stressors at increased rates or unique stressors when compared with other caregivers or the general population. Compared to non-kinship caregivers, kinship caregivers could face greater financial strain and limited support avenues [54]. This trend is concerning, especially for informal kinship caregivers who may not receive the same help from social services as those who undergo formal care arrangements and traditional foster caregivers [52,54,55].

Stress experienced by kinship caregivers may adversely influence the quality of care they provide to their children and/or children’s outcomes. Washington et al. (2013) [56] found higher academic competence among children whose caregivers had lower stress levels. Kinship caregiver mental health could also impact social, behavioral, and/or emotional outcomes for children. When comparing children in kinship care with children in nonrelative foster care, Garcia et al. (2015) [57] found that positive social, behavioral, and emotional outcomes were better among kinship care children, but these outcomes were primarily observed among children whose caregivers were never depressed or had reduced their depression over time.

Stressors related to inadequate support and limited resources significantly affect African American kinship care families. Several studies have found that African American kinship caregivers face difficulties accessing social services, encounter barriers in securing support for their children’s educational needs, possess scarce financial resources, and receive insufficient family support—sometimes in comparison to caregivers from other family types [10,58,59]. However, further research is needed to identify stressors unique to African American kinship care households and to enable more precise comparisons with kinship care households from other populations.

### 1.6. Relationship with Birth Parents

Kinship care arrangements could present more opportunities for birth parent involvement with parenting than with traditional nonrelative foster care arrangements, given that relationships often exist between the children, birth parents, and caregivers prior to kinship care arrangements [60]. Birth parent involvement with children in kinship care may depend on the circumstances leading to their placement in kinship care and the terms and conditions of kinship care arrangements [61]. Additionally, the type of kinship care placement (formal vs. informal) likely influences birth parent involvement, as informal arrangements typically have fewer formal restrictions concerning birth parent contact with the kinship care family [61,62]. Another important factor is the quality of interactions between kinship caregivers and birth parents. Higher levels of birth parent involvement are often observed when positive relationships exist between birth parents and caregivers, whereas conflict between the two parties may diminish parental involvement [63]. Moreover, the quality of these relationships can also have a positive impact on children’s outcomes [62].

According to previous research, positive relationships between children and birth parents can promote the development of children in kinship care households. For example, competence levels appear higher among African American kinship care children who have better relationships with their birth fathers and mothers [56,62]. Other studies have indicated the importance of the relationship between children in kinship care and their birth fathers [62,64]. Gable et al. (2024) [65] found that, in a Midwest sample of almost 200 children, those with weekly or monthly contact with their birth fathers, as opposed to no contact, had a lower likelihood of learning disabilities, hyperactivity disorders, and/or mental health issues.

Parental involvement by children’s biological mothers and fathers can impact caregivers’ stress positively or negatively, and this can, in turn, influence children’s outcomes. Washington et al. (2024) [66] proposed that caregiver stress may be heightened when kinship caregivers experience conflict with children’s birth parents. Additionally, challenges from birth parents may be more apparent among African American kinship caregivers compared to other caregivers [54,58]. For example, in a mixed sample of African American and White caregivers, Smith-Ruiz et al. (2021) [58] found that African American caregivers faced less cooperation, less support, and a greater lack of involvement from children’s birth parents than their White counterparts.

Continued investigation into birth parent involvement is essential to understanding its direct influence on the mental health and overall well-being of children residing in kinship care. Addressing this gap can inform interventions aimed at improving depression and anxiety outcomes. Such research is particularly critical for African American families, who are disproportionately represented in kinship care arrangements.

## 2. Methods and Results

This mixed-methods study explored the relationships between caregiver stress and the quality of birth mother–child relationships and child depression and anxiety. Our hypotheses and research questions follow.

### 2.1. Hypotheses

1. Caregivers with lower stress levels will have children with lower depression and anxiety levels.
2. The more positive birth mothers’ relationships with their children are, the lower their depression and anxiety levels.

### 2.2. Research Questions

a. What were caregivers’ experiences with stress related to kinship care as well as stress in general?
b. What were caregivers’ perceptions of birth mothers’ relationships with children?

This mixed-method study used a sequential explanatory design in which the quantitative data collection and analysis were conducted first, followed by the qualitative phase. The qualitative component is intended to explain, interpret, and expand the quantitative results [67]. This sequential explanatory design is ideal when researchers want to identify and understand patterns, relationships, or factors that require deeper understanding and exploration. In this type of design, preference is given to the quantitative component, with the qualitative component playing more of a supporting role. The integration of both components occurs during the interpretation and discussion of qualitative findings, intended to illuminate and highlight the quantitative results [68].

### 2.3. Phase 1: Quantitative Component

#### 2.3.1. Participants

Participants for this study were adults over 18 who identified as African American and were currently raising related children aged 5–12 in formal or informal kinship care arrangements [22].

They resided in Guilford and Rockingham counties or surrounding areas. Inclusion criteria were applied to ensure the sample’s relevance and representativeness, excluding caregivers of other racial backgrounds or those outside the defined geographic region. Guilford and Rockingham counties were selected based on their inclusion in the pilot study and their sizable African American populations; this study was not designed for generalization through random sampling but to explore targeted experiences within these communities. Recruitment involved distributing flyers and advertisements in high-traffic community spaces such as local agencies, schools, and family-serving organizations. We also partnered with community stakeholders and agencies that support kinship families to conduct direct outreach and build trust.

All participant data were anonymized and securely stored to maintain confidentiality, and Institutional Review Board (IRB)-approved protocols were followed throughout the recruitment and data collection processes. These measures ensured ethical and robust data collection to explore the lived experiences and outcomes of African American kinship families. Sample descriptives are provided in Table 1. A total of 58 interviews were conducted from 45 households, 13 of which provided data for two children. Of those 58 interviews, 52 contained child-level outcome data from the Behavior Assessment System for Children (BASC-3) Anxiety subscale, and 50 contained data from the BASC-3 Depression subscale. Demographic data were also available from the additional interviews, resulting in sample sizes for child-level demographic characteristics ranging from 54 to 58.

#### 2.3.2. Phase 1: Quantitative Measures

Measures for the dependent variables (children’s depression and anxiety) and the main independent variables (caregivers’ stress and birth mother–child relationship) have acceptable reliability and validity, as reported in multiple studies.

Dependent Variables. To assess children’s depression, we used the Parent Rating Scales of the Behavior Assessment System for Children (3rd ed., BASC-3) [69]. This BASC-3 measure was also used to assess children’s anxiety levels. Caregivers answered questions about how often children exhibited certain behaviors, such as “worries about things that cannot be changed” or “cries easily,” and responses were captured on a 4-point scale (1 = “never” to 4 = “almost always”). The Parent Rating Scale for the BASC-3 has reliability coefficients, with Cronbach’s alphas ranging from 0.83 to 0.89 for Anxiety and 0.86 to 0.90 for Depression, and reliability is generally consistent between males and females [69]. Analyses of factor structure for the subscales, correlations with various assessments, and clinical group score profiles have verified the validity of this instrument [69].

Main Independent Variables. Caregivers’ stress levels were assessed using the 36-item Parenting Stress Index (PSI)–Short Form [70]. Cronbach’s alphas for the PSI indicate high reliability for each subscale—0.88 for both Parental Distress and Parent–Child Dysfunctional Interaction, 0.89 for Difficult Child, and 0.95 for Total Stress [71]. Construct and convergent validity for the PSI were verified by regression analyses [71,72]. Caregivers answered questions such as “I feel trapped by responsibilities as a parent,” and responses were captured on a 5-point scale (from “strongly agree” to “strongly disagree”). Sums of total scores are calculated, and higher scores indicate higher stress levels.

The birth mother–child relationship was assessed using the Child–Parent Relationship Scale Short Form [73]. This 15-item measure assesses perceptions of closeness, interactive behaviors, communication, and conflict between children and their birth mothers. Responses are captured on a 5-point scale (1 = “definitely does not apply” to 5 = “definitely applies”). For the Child–Parent Rating Scale, Cronbach’s alphas ranged from 0.78 to 0.84 for parental conflict and from 0.69 to 0.74 for parental closeness [73].

Other Independent Variables. Healthy family functioning was assessed using the Beavers Self-Report Family Instrument [74,75]. Caregivers answered questions from this instrument about family competence regarding how well family units carry out organizing and nurturing functions. The extent of family resources within households was assessed using the Family Resource Scale [76]. This instrument was used to assess the adequacy of resources such as food, money for necessities, and housing conditions. Given the historical hardships of African American families in the United States, the Cultural and Racial Experiences of Socialization scale (CARES) [77] assessed the frequency of racial and ethnic socialization messages in households. Concepts examined through this instrument include cultural legacy, cultural pride, racial and religious coping, interracial coping, alertness to racism, and internalized racism.

Covariates. We used the following demographic covariates in our analyses: child age, child gender, caregiver’s age and annual income, type of kinship care, and children’s length of stay in kinship care. Child age and caregiver’s age were recorded in years and months. Child gender was dummy coded as male (0) and female (1). Caregivers’ income was recorded in US Dollar categories from “4999 or less” to “50,000 or more.” The type of kinship care was dummy coded as formal kinship care (0) and informal kinship care (1). Length of stay was recorded in years and months. All models controlled for these covariates.

#### 2.3.3. Phase 1: Data Analysis

Data management and preliminary analyses were carried out using R v4.4 [78], and modeling was conducted using Mplus v.8 [79]. Relevant characteristics of the child (i.e., sex and age), caregiver (i.e., age, education, income, and employment status), and environment (i.e., length of time in KC, household stress) were examined using descriptive analyses. General linear modeling was employed to estimate associations between caregiver stress, healthy family functioning, family resources, birth mother–child relationship, and CARES subscales and measures of children’s behavioral outcomes (i.e., anxiety and depression). Some missingness and minimal nesting (i.e., two children within a household) were present in the data. Therefore, full information maximum likelihood estimation was used to retain cases with partial data, and robust standard errors were estimated to accommodate nonnormality and nonindependence of outcomes.

For ease of interpretation, item means rather than sums were used for all total and subscale scores. Caregiver age was scaled to 5-year units, and length of stay in kinship care was scaled to 6-month units. Finally, all covariates were grand mean–centered before model entry. Significant collinearity between several CARES subscales was identified, and problematic subscales were removed. Specifically, the Cultural Legacy (CL) and Racial and Religious Coping (RRC) with antagonism subscales were highly correlated (*r* > 0.85) with the Cultural Pride (CP) reinforcement subscale and with each other (*r* = 0.84). Furthermore, CP accounted for the majority of variance in outcomes otherwise accounted for by CL and RRC, while itself accounting for the most unique variance in outcomes. Similarly, the Promotion of Mistrust (PM) and Internalized Racism (IR) subscales were highly correlated (*r* = 0.82) and accounted for a similar pool of variance in outcomes. IR contributed the most to the variance in outcomes; thus, IR was retained. Supplementary tables are provided in Supplementary Materials for each CARES subscale removed from the final models (Tables S1–S6).

### 2.4. Phase 1: Quantitative Results

#### 2.4.1. BASC Depression

A total of 50 participants provided data for the BASC Depression Scale. Model results for depression (Table 2) indicated that the birth mother–child relationship was significantly and negatively associated with reported depression (β = −0.132, *SE* = 0.056, *p* = 0.019). Among CARES subscales, only Alertness to Racism was significantly associated with depression (β = −0.256, *SE* = 0.101, *p* = 0.011). Consistent with results for anxiety (Table 3), Caregiver Stress was also positively associated with depression (β = 0.310, *SE* = 0.089, *p* = 0.001). Lastly, Caregiver Age (β = −0.052, *SE* = 0.025, *p* = 0.039) was also significantly predictive of reported levels of anxiety in children (Table 2). Overall, the model accounted for approximately 51.1% of the variance in self-reported depression ratings.

#### 2.4.2. BASC Anxiety

A total of 52 participants provided data for the BASC Anxiety Scale. Unlike depression, model results regarding anxiety (Table 3) indicated that the birth mother–child relationship was not significantly associated with reported anxiety (β = −0.025, *SE* = 0.053, *p* = 0.631). Among CARES subscales, Cultural Pride was positively associated (β = 0.241, *SE* = 0.104, *p* = 0.021) and Alertness to Racism was negatively associated with reported anxiety (β = −0.378, *SE* = 0.163, *p* = 0.021). Interracial Coping was not significantly associated with anxiety (*p* = 0.21). Lastly, Caregiver Stress was also positively associated with reported anxiety levels (β = 0.357, *SE* = 0.140, *p* = 0.011). Overall, the model accounted for approximately 55.4% of the variance in self-reported Anxiety ratings.

### 2.5. Phase 2: Qualitative Component

#### 2.5.1. Thematic Analysis

Thematic Analysis (TA) as a qualitative methodology provides a rich and compelling view into the lives, experiences, and perspectives of kinship caregivers [80]. Thematic Analysis (TA) is a widely used qualitative research method that identifies, analyzes, and reports patterns and themes within data. TA is ideal because it can be used with different research paradigms [80,81]. TA allows researchers to explore individual experiences to analyze social constructs. Moreover, TA allows for an in-depth exploration of the explicit and implicit meanings in participants’ responses, ensuring that the analysis is deeply rooted in the data while also being responsive to the broader theoretical and social contexts relevant to the research question [81,82,83]. TA is an ideal choice for addressing complex, multifaceted phenomena in qualitative research, such as examining how kinship care affected children’s outcomes [80,81,83].

#### 2.5.2. Qualitative Participants

Sixteen kinship caregivers from the quantitative sample also completed qualitative interviews, of whom fifteen identified as female and one as male. Eleven were single, widowed, or divorced; five were married, although spousal caregiving involvement varied. Households included 1–3 children aged 2–16, with some also supporting adult children or other relatives. Caregiver–child relationships included grandchildren, nieces/nephews, great-nieces, second cousins, and paternal nieces. Kinship placements stemmed from issues such as parental substance abuse, incarceration, neglect, domestic violence, mental illness, abandonment, termination of parental rights, death, or Child Protective Services intervention.

Custody statuses ranged from full guardianship to informal caregiving without legal recognition. Children often had trauma histories (e.g., exposure to domestic violence, drug use, neglect, human trafficking) and behavioral/emotional challenges such as ADHD, PTSD, depression, and anxiety. Some, however, thrived with consistent emotional and educational support. Financial resources varied—some caregivers received child support, Social Security, or kinship assistance, while others relied on fixed incomes, part-time work, or retirement benefits. Financial strain was common, especially without formal aid. Support from extended family ranged from robust to minimal.

#### 2.5.3. Data Collection

The names of those who agreed to participate in the qualitative component were given to a member of our research team, who then contacted them to see if they were still interested in participating and scheduling an interview. All interviews took place via phone or Zoom. Interviews lasted from 30 min to 1 h. They were recorded via Zoom or a digital recorder and transcribed by a transcription company. The interview guide had several domains, asking participants questions about coparenting and parental involvement, birth parent and child relationship, caregiver and child relationship, kinship family functioning, kinship family resources, self-care, and how caregivers practice regarding racial socialization.

#### 2.5.4. Phase 2: Data Analysis

Thematic analysis was used as the primary method for analyzing qualitative data due to its practicality and systematic nature. This approach, as outlined by Braun and Clarke (2006) [81], involves six distinct yet iterative phases, ensuring a rigorous and transparent process. The first phase, familiarizing with the data, involved thoroughly reading and re-reading transcripts to immerse oneself in the content. Next, initial codes were generated, capturing key features across the dataset. In the third phase, these codes were grouped into potential themes that represented broader patterns of meaning. Themes were then reviewed and refined in phase four to ensure they accurately captured the data and were distinct from one another. During phase five, themes were defined and named, creating clear and concise descriptions that highlighted their essence. Finally, the sixth phase involved producing the final report, which synthesized the analysis into a coherent and compelling narrative, grounded in a rich, nuanced interpretation of the data. This structured yet flexible approach facilitated a comprehensive exploration of the dataset, providing insights into the lived experiences of participants [81,83].

We determined that thematic saturation was reached when no new themes, codes, or patterns emerged from the interviews, and participant responses began to repeat previously identified concepts across domains [81,84]. After approximately 14 interviews, the research team observed strong redundancy in key themes such as caregiver stress, financial strain, and dynamics of birth parent involvement. Two additional interviews were conducted to confirm saturation and ensure that any divergent experiences were captured. In addition, our sample size of 16 participants aligns with qualitative research standards for achieving thematic saturation in studies with focused research questions and relatively homogeneous populations [84]. While saturation was the guiding principle, we also aimed for an adequate sample size to enhance confidence in the credibility and transferability of findings.

To ensure the rigor of qualitative findings, we followed Braun and Clarke’s (2006) [81] six-step thematic analysis process. Two researchers independently coded transcripts and compared results to ensure consistency. A codebook was developed early and iteratively refined to maintain coding uniformity. Discrepancies were resolved through discussion until consensus was reached. We maintained an audit trail of coding decisions and analytic memos to enhance transparency. Peer debriefing with senior researchers provided an additional layer of review for theme interpretation. These strategies support the credibility, dependability, and confirmability of the findings [84,85].

#### 2.5.5. Phase 2: Qualitative Results

The following overarching themes emerged from the qualitative analysis, reflecting the complexity of kinship care: (1) the weight of kinship care—navigating emotional, behavioral, and structural challenges, and (2) birth parent relationships—a continuum of connection, conflict, and loss. These themes illuminate the multidimensional burdens caregivers shoulder—including persistent grief, behavioral crises, and systemic inequities—while revealing how birth parent relationships shape children’s adjustment through patterns of support, disruption, or ambiguity. Together, these findings move beyond generalized notions of caregiver stress to underscore the intersecting emotional and structural forces that define kinship care experiences and the implications for trauma-informed, culturally responsive interventions.

#### 2.5.6. Weight of Kinship Care: Navigating Emotional, Behavioral, and Structural Challenges

Kinship caregivers described their roles as far more complex than traditional parenting, requiring them to manage cumulative burdens that were emotional, behavioral, and structural in nature. Beyond supporting children through trauma and loss, caregivers often navigated their own grief, addressed challenging behaviors, and coped with persistent financial strain—frequently without adequate systemic support. These experiences reveal patterns that extend beyond general caregiving stress, shaped by race, cultural expectations of family obligation, and structural inequities embedded in child welfare systems. The interplay of grief, behavioral crises, and economic hardship created layers of cumulative disadvantage that intensified caregiver burnout and heightened risks to children’s emotional well-being [86]. These findings underscore the urgent need for trauma-informed, culturally responsive, and structurally equitable interventions to sustain kinship caregivers and promote child stability.

##### Persistent Grief and Emotional Strain

Caregivers frequently spoke of the emotional toll of parenting children who were grieving, even as they managed their own sense of loss. Edward Ferguson, an uncle caring for his niece and nephew, reflected, “It breaks my heart for my sister. I think they still grieve to a certain extent. Both of them have cried when they left… I don’t think they’re done grieving.” Molly Evans, a grandmother caring for her grandson, described how her grandson struggled to stay present in school due to the loss of his mother, saying, “Sometimes he’d be spaced out. He’d say—you know—I can’t see—you know—I think about my mom, and I don’t have nothing else on my mind. I can’t focus on my class.” Other caregivers described how grief resurfaced when birth parents made unexpected appearances. Brenda King, a grandmother raising her granddaughters, explained, “To be honest with you, it’s like we get in a good spot. And then she’ll [birth mother] come, and it’s almost like we’re starting over again.” Even when children outwardly appeared to be functioning well, their ongoing grief deeply impacted the emotional climate of the household and contributed to caregiver exhaustion.

Caregivers described grief as a persistent, cyclical stressor that resurfaced when birth parents reappeared unexpectedly. Unlike the linear grief models often assumed in child welfare practice, these narratives suggest that ambiguous loss destabilizes household equilibrium [87]. This finding extends existing research by illustrating how kinship caregivers navigate repeated grief activation, emphasizing the need for interventions that support both children and caregivers through ongoing, unpredictable loss.

##### Managing Behavioral Crises and Mental Health Needs

Caregivers often faced behavioral challenges that stemmed from children’s trauma and the instability of their relationships with biological parents. Linda Taylor, a great-aunt raising two nieces, recounted how a visit with their mother led to behavioral regression: “She told the oldest one that she didn’t have to listen to me… When we got back to North Carolina, that’s exactly what she did. She was being disrespectful, getting suspended… I finally sat her down and talked to her. She said, ‘Well, my mom told me I don’t have to listen to you.’ It has gotten better because I stayed firm.” Other caregivers described how visits or even brief contact with a parent triggered outbursts or defiance. Kennedy Isaacs, a grandmother caring for her grandson, shared how overwhelmed she became caring for her grandson: “It just took such a hold on me… my other kids was telling me—you got to do something. You got to give them back.”

Mental health challenges further complicated caregiving. Lisa Taylor, an aunt raising her niece, shared that her older niece, still affected by her mother’s words, was diagnosed with ADHD, mood swings, and depression. Samantha Thomas, a great-aunt raising her great-nieces, described how her niece’s anxiety manifested in self-harm: “She used to be real nervous… the police was always involved… she used to pull her hair out because her nerves were so bad.” These mental health struggles required patience, structure, and emotional labor, contributing to the cumulative stress caregivers experienced.

These narratives reveal that caregiving demands extended beyond discipline into crisis management, with caregivers serving as emotional anchors in the absence of consistent professional support. Unlike traditional foster care, many informal kinship caregivers lacked access to specialized mental health services, leaving them to navigate their children’s trauma and mental health needs without adequate guidance. The interplay of behavioral disruption, unresolved grief, and a lack of systemic support amplified caregiver stress—an observation that complements the quantitative finding that higher caregiver stress correlates with children’s depression and anxiety.

##### Financial Hardships and Structural Inequities

In addition to emotional and behavioral stress, kinship caregivers often faced persistent financial strain, particularly older adults or those living on fixed incomes. Henrika Thomas, who raised three granddaughters and an adopted daughter with cerebral palsy, shared, “I work—I have a home childcare business, and I do therapeutic foster care,” yet she received less financial support than licensed foster parents. Wendy Newman, a grandmother raising her granddaughter, echoed the economic hardship: “I’m on a fixed income… I’ll try to work a part-time job every now and then. But yeah, I’m on a fixed income. But I’ve been doing this with this all my life.” Caregivers juggled employment, household expenses, and legal responsibilities while lacking consistent support, highlighting the inequities they faced within the caregiving system.

These accounts illuminate a critical structural inequity: the caregiving system often assumes that kin will “step in” without providing comparable supports available to foster families, despite the fact that kinship caregivers frequently assume these roles unexpectedly and without preparation. This expectation reflects broader policy gaps rooted in systemic bias, where family obligation substitutes for institutional responsibility.

The long-term nature of kinship care amplifies these challenges. Unlike short-term foster placements, many kinship arrangements last for years, requiring sustained financial resources for education, healthcare, and legal expenses. For older caregivers living on retirement or disability income, these costs create persistent vulnerability and emotional strain. This theme highlights the pressing need for policy reforms that provide equitable financial support to kinship caregivers, particularly those in informal arrangements, to prevent family instability and child displacement.

#### 2.5.7. Birth Parent Relationships: A Continuum of Connection, Conflict, and Loss

Caregivers described birth parent relationships as a defining influence on kinship care—shaping children’s sense of belonging, emotional regulation, and behavioral outcomes. These relationships existed along a continuum rather than a simple presence-or-absence dichotomy. At one end, consistent connections acted as a protective factor, offering children emotional continuity despite parental absence from daily care. At the other end, undermining and hostile dynamics created loyalty conflicts and behavioral disruption, intensifying caregiver strain. Between these extremes, many families experienced ambiguous loss and relational uncertainty, where parents were physically present but emotionally unavailable or permanently changed, leaving children with grief that lacked closure. This continuum reflects the interplay of attachment disruption, ambiguous loss, and systemic inequities, highlighting the need for trauma-informed, culturally responsive interventions that support safe parental contact when appropriate and equip caregivers to navigate complex relational dynamics.

##### Consistent Connections as a Protective Factor

Some caregivers described biological parents who remained present in their children’s lives despite being unable to provide full-time care. These parents often stayed connected through phone calls, birthday celebrations, and attendance at major events. Kris Cameron, a grandmother raising two granddaughters, observed, “Their mom always stayed connected. She called them. She never forgot birthdays. She never forgot Christmas. And in terms of them going to live with her solely, that would be a challenge… They love their mother… there’s always a sadness that that leaves.” Children’s reactions to their parents varied based on age and experience. Linda Sullivan noted, “My grandson loves his mother… when she comes around, it just lights up his day. But my granddaughter feels different… she’s been with her longer, seen—maybe more and remembers more.” Some children found comfort in the memory of their parents or tokens of the past, even while grappling with the pain of separation. Wendy Newman, a grandmother caring for her granddaughter, described how she still clung to the image of her childhood bedroom, finding security in those memories. Over time, many children experienced emotional healing, but the path remained uneven.

Such efforts provided emotional continuity and reinforced children’s sense of belonging, mitigating feelings of abandonment and fostering resilience. These examples suggest that structured opportunities for positive parental involvement—when safe and feasible—may buffer children from the most severe emotional consequences of separation.

##### Undermining and Hostile Dynamics

Not all biological parent relationships were benign. Some were deeply strained or adversarial, with birth parents actively undermining caregivers’ authority. Samantha Thomas, who cared for her nephew and two great-nieces, shared, “She [biological mother] has disrespected me plenty of times on social media. She has called social services to my home more than once… It’s like I’m the most hated when I was all I was trying to do was help out.” In more severe cases, caregivers supported children through the death of a parent. Opal Krenshaw, a first cousin raising her second cousins, recounted how the children found their mother after she overdosed: “They go in and find their Mama had OD. They called 911.” Children who lost parents often exhibited sadness, anger, or emotional shutdowns. Wendy Newman’s granddaughter struggled with waves of frustration. “Sometimes, she just comes in there where I’m at, and I just hold her and talk to her and tell her it’s going to be all right… I can’t erase the pain… but I can always kind of uplift her a little bit.” Some children were abandoned rather than bereaved. Holly Nelson, an aunt raising her niece, explained how her brother chose not to be involved in his daughter’s life and reflected on the mother’s parenting challenges: “She emancipated at 14 or 15… There’s a lot of brokenness there.”

These narratives illustrate ambiguous loss [87], where the parents’ physical absence or emotional unavailability creates a chronic sense of instability. Unlike discrete bereavement, ambiguous loss lacks closure, making coping more complex for both children and caregivers.

Together, these accounts highlight the emotional labor caregivers invest in buffering children from trauma while managing their own grief. They underscore the need for interventions that support caregivers in addressing compounded loss and equip them with strategies to navigate hostile or absent parental relationships, including trauma-informed counseling, structured visitation plans, and systemic advocacy.

##### Ambiguous Loss and Relational Uncertainty

Other relationships were more ambiguous. Some children struggled to make sense of emotionally or physically unavailable parents. Edward Ferguson, an uncle caring for his niece, shared that although his sister survived a serious accident, she was no longer the parent her children remembered: “It breaks my heart for my sister… life changes in an instant.” This created confusion and unprocessed grief in the children, who did not understand why their mother could no longer care for them.

Bae Benson, caring for her 8-year-old cousin, described how Kwame internalized his pain: “Although Kwame is very smart, very wise, he also is very delayed mentally… he shuts down a lot when it comes to like being able to express feelings… he doesn’t cry hardly ever… he’ll fight his tears.” For children facing ambiguous loss, caregivers often became emotional anchors, patiently guiding them through their pain even when words failed.

For caregivers, these situations required profound emotional labor. They acted as emotional anchors, sustaining children through long-term uncertainty without access to specialized support systems. Unlike situations of parental death, which typically trigger formal grief resources, ambiguous parental loss often goes unacknowledged, leaving caregivers and children to cope in isolation.

This theme underscores the need for trauma-informed and grief-responsive interventions tailored to the unique realities of kinship care. Services should include counseling for both children and caregivers, as well as guidance for navigating relational boundaries when parental presence is inconsistent or compromised. Such supports are essential for preventing the compounding effects of unresolved grief on children’s behavioral health and caregivers’ well-being.

## 3. Discussion

This mixed-methods study examined the impact of caregivers’ stress and the quality of birth mother–child relationships on child depression and anxiety symptoms among African American children who reside in kinship care. We acknowledge the importance of using lived experience to understand the origins of the quantitative results. By integrating quantitative and qualitative approaches, we developed a comprehensive understanding of these contributing factors on children’s outcomes.

### 3.1. Quantitative Results

Our first hypothesis proposed that caregivers with lower levels of stress would have children exhibiting lower levels of depression and anxiety. This hypothesis was supported, as findings indicated that reduced stress among kinship caregivers was associated with decreased symptoms of depression and anxiety in children. These results are similar to prior research demonstrating the detrimental impact of caregivers’ stress and mental wellness on child mental health and overall well-being [88,89,90,91]. However, the current study extends this body of knowledge by specifically examining depression and anxiety symptoms within African American formal and informal kinship care families, which are often underrepresented in research, despite being disproportionately represented among kinship care families.

Several studies have documented elevated stress levels among individuals raising children in kinship care [51,52,53], and our current study also found elevated stress levels for these individuals. Researchers have suggested that factors (e.g., financial strain, limited social support, and caregiving burden) contribute to heightened stress within this population [53,59,92,93,94]. Given that inadequate resources are a major source of caregiver stress, it is reasonable to infer that these conditions negatively affect child outcomes. For instance, caregivers under financial strain may be unable to afford mental health services for their children, leading to unmet needs and exacerbation of symptoms. Moreover, ongoing stress in the caregiving environment may limit caregivers’ emotional availability, reduce their capacity to engage in responsive parenting, and increase the likelihood of contributing to children’s psychological distress [95,96,97,98]. The findings from this first hypothesis highlighted the importance of providing targeted support services to caregivers to reduce their stress and ultimately promote positive mental health outcomes for children in their care.

Our second hypothesis was that more positive relationships between birth mothers and their children would be associated with lower levels of depression and anxiety in the children. Findings partially supported this hypothesis, indicating that the quality of the birth mother–child relationship plays a protective role in children’s mental depressive symptoms. This result is consistent with previous research in the general population, showing that warm, supportive, and responsive parenting is linked to a reduced risk of internalizing symptoms in children [99,100,101]. Additionally, there is a limited body of research focusing specifically on parental involvement within kinship care families [62,65,102]. For example, Washington et al. (2014) [62] is one of the few studies in this area that found positive relationships between African American birth parents and children were associated with more favorable child outcomes. Thus, our current study adds to this area of research.

Interestingly, for this study, there was no statistical significance relationship between the birth mother–child relationship and children’s anxiety. This finding suggests that the emotional quality of this primary attachment may play a more direct role in shaping children’s internalized feelings of sadness, hopelessness, and worthlessness—hallmarks of depression—rather than the hyperarousal and worry characteristic of anxiety. Theoretically, attachment and loss models suggest that disruptions or ongoing emotional ambivalence in the birth mother–child relationship can lead to unresolved grief or chronic insecurity, particularly in children who remain near but are no longer in the custody of their birth mothers [60].

It appears that positive relationships among birth mothers and children may foster a sense of emotional security, self-worth, and resilience, all of which serve as a buffer against the development of depression [103]. In contrast, disrupted or conflictual maternal relationships may increase vulnerability to psychological distress. This study’s findings underscore the importance of strengthening the birth mother–child bond, even if they are not the primary caregivers, to reduce symptoms.

### 3.2. Qualitative Results

The qualitative findings of this study illuminate the following interconnected themes that shape the lived realities of African American kinship caregivers: the weight of kinship care—navigating emotional, behavioral, and structural challenges; and birth parent relationships—a continuum of connection, conflict, and loss. These themes move beyond generalized notions of caregiver stress to expose the cumulative and systemic nature of the challenges caregivers face, while also revealing the profound relational dynamics that influence children’s well-being and family stability. Taken together, these findings highlight how caregiving in kinship contexts is simultaneously an act of love and resilience and a site of vulnerability shaped by trauma, inequity, and complex family ties.

The weight of kinship care reflects the multifaceted and enduring responsibilities that caregivers assume, extending far beyond the scope of traditional parenting. These caregivers were not simply raising children; they were managing grief, trauma, and chronic uncertainty in environments often lacking adequate systemic support. Emotional strain was a recurring theme, with grief emerging as cyclical rather than linear. For many, the sporadic reappearance of birth parents reignited old wounds, destabilizing household routines and emotional equilibrium. This pattern resonates with theories of ambiguous loss [87], where the absence of closure makes grief an ongoing process rather than a discrete event. Alongside emotional labor, caregivers reported significant behavioral challenges rooted in children’s exposure to trauma and adverse childhood experiences. These behaviors, coupled with mental health diagnoses such as ADHD, anxiety, and mood disorders, required caregivers to act as crisis managers and emotional anchors without consistent access to trauma-informed services. The weight of these responsibilities was intensified by structural barriers. Financial hardship was nearly universal among participants—particularly older caregivers on fixed incomes—and compounded by systemic inequities that assume kin will step in without sufficient institutional or financial support. This expectation reflects a broader policy gap rooted in cultural assumptions about family obligation, which too often substitutes for systemic responsibility. These findings underscore the urgent need for structural interventions, including equitable financial resources, caregiver training, and the integration of trauma and grief-informed care into kinship support services.

Birth parent relationships constituted the second major theme, emerging as a continuum of connection, conflict, and loss. These relationships carried profound implications for children’s emotional security and behavioral adjustment. At one end of the spectrum, consistent parental involvement—even when limited—provided emotional continuity, mitigating feelings of abandonment and reinforcing a sense of belonging. At the opposite end, some caregivers navigated highly adversarial dynamics, with birth parents actively undermining their authority, creating loyalty conflicts, and triggering behavioral regression in children. Between these poles lay the complexity of ambiguous loss, where parents were physically present but emotionally unavailable or permanently changed by illness, addiction, or trauma. These circumstances left children grappling with grief without closure, identity confusion, and an enduring sense of instability. For caregivers, these relational dynamics amplified stress, requiring not only emotional labor but also strategic negotiation to maintain stability in the face of unpredictable parental involvement. These findings suggest the need for targeted interventions that support children in processing ambiguous and traumatic loss, equip caregivers with strategies for navigating loyalty conflicts and relational boundaries, and establish structured opportunities for safe, developmentally appropriate parental contact when feasible.

Together, these themes underscore the dual reality of kinship caregiving—a profound act of resilience that sustains family bonds, and a site of structural neglect where caregivers shoulder immense burdens without adequate support. The findings affirm the critical importance of trauma-informed, culturally responsive interventions tailored to the unique context of African American kinship care. Such interventions must operate at multiple levels—individual, familial, and systemic—to address the intersecting emotional, relational, and structural challenges that define these caregiving experiences. Financial and legal support must be equitable for informal kinship caregivers to reduce chronic stress and prevent placement disruptions. In addition, mental health services that are both accessible and culturally grounded are essential to address children’s behavioral and emotional needs and to sustain caregiver well-being. By centering these realities, child welfare systems can move toward a more just and responsive model of care—one that honors the resilience of kinship caregivers while dismantling the systemic inequities that undermine their efforts.

### 3.3. Strengths and Weaknesses of the Study

There are several strengths and limitations to consider when interpreting the results of this study. First, this study is among the few that examines kinship care using a mixed methods design, integrating both quantitative and qualitative data to explore how caregiver stress and the birth mother–child relationship relate to child depression and anxiety. This is an underexplored area in the literature. Second, the study includes two populations often excluded from kinship care research—informal kinship care families and birth parents. Most existing studies focus on formal kinship care families under the supervision of the child welfare system, despite informal arrangements accounting for approximately 90 percent of all kinship care families [9]. Third, because African American families are more likely than other racial groups to participate in kinship care, this study helps expand the literature on this population, where additional research is needed. By including populations often overlooked in prior studies, this research contributes to a more comprehensive understanding of kinship family dynamics and support needs to improve children’s depressive and anxious symptoms.

In light of these strengths, when interpreting the results of the study, there are several limitations to consider. First and foremost, this study’s findings rely only on caregiver reports for quantitative measures and qualitative exploration, which may introduce reporting bias, particularly given that caregivers may underrecognize or unintentionally minimize internalizing symptoms like depression and anxiety. Second, this study is not generalizable due to its focus on kinship care families from a specific region of the United States and a modest sample size. Third, this study did not include birth father involvement, which limits the understanding of the full scope of ways parent–child dynamics influence children’s depression and anxiety.

## 4. Implications for Practice and Research

Findings from this study highlight the strong connection between caregiver stress and child mental health outcomes in African American kinship care families. Specifically, higher caregiver stress was associated with higher levels of depression and anxiety in children. These results underscore that children’s mental health and well-being cannot be fully understood or addressed without also attending to the stress levels and well-being of their caregivers. In practice, interventions should focus on supporting caregivers in managing and reducing stress. Strategies may include promoting physical activity, connecting caregivers with peer support groups, and helping them navigate complex systems such as schools, healthcare, and financial assistance programs. Practitioners working with kinship families should be trained to recognize signs of caregiver stress and offer culturally appropriate tools and referrals tailored to the needs of African American caregivers. Reducing caregiver stress most likely will lead to improved mental health outcomes for the children in their care.

This study also found that more positive relationships between children and their birth mothers were associated with lower levels of child depression, though not anxiety. These findings suggest that appropriate practitioners should consider family-based interventions such as family therapy or facilitated visitation. Supporting the birth mother–child relationship can be important for children’s mental health and long-term adjustment. In cases where reunification is a goal, families and professionals should work collaboratively to assess readiness and identify the supports needed to achieve safe and stable reunification. These conversations should also consider the reasons for the original placement in kinship care and how they may continue to affect the family system.

Implications for future research include the need to expand knowledge on the role of birth fathers in kinship care and their potential impact on children’s mental health. Fathers are often underrepresented in kinship care research, despite the limited evidence that paternal involvement can be important for children’s outcomes. In general, future studies should explore ways to include both mothers and fathers in family-centered interventions, while minimizing added stress for kin caregivers who may already be overextended.

Additionally, research should examine other family characteristics that may contribute to positive child mental health outcomes, such as household stability or extended family support. The findings from this study can serve as a foundation for developing and testing culturally appropriate interventions designed specifically for African American kinship care families. Such research will be critical for informing policies and practices that promote resilience and positive outcomes for children.

## 5. Conclusions

In sum, it is important to examine the impact of caregivers’ stress and the quality of birth mother–child relationships on depression and anxiety symptoms among African American children residing in kinship care. Despite facing numerous stressors, African American kinship caregivers continue to care for their children with love and intentionality and often facilitate birth parent involvement. Recognizing how caregiver stress and birth parent involvement influence the mental health and overall well-being of children in kinship care can encourage child welfare stakeholders and other advocates to support and promote further research in this critical area.

## Figures and Tables

**Table 1:** Demographic table.

Variable (*n* = 58)	Mean	Median	Range
Child’s age in years	9.11	9.08	4.25–12.92
Number of children per household	2.51	2	1–6
Length of child’s stay with caregiver/months	66.09	61.00	5–144
Variable	*n*	%[i]	
Child’s gender			
Female	38	66.67	
Male	19	33.33	
Caregiver’s annual income (US Dollars)			
4999 or less	2	3.45	
5000–9999	2	3.45	
10,000–14,999	10	17.24	
15,000–19,999	5	8.62	
20,000–24,999	6	10.34	
25,000–29,999	10	17.24	
30,000–34,999	4	6.90	
35,000–39,999	1	1.72	
40,000–44,999	3	5.17	
50,000 or more	15	25.86	
Education			
No high school diploma/GED	2	3.45	
High school graduate	6	10.34	
Some college or trade school	30	51.72	
College graduate	13	22.41	
Graduate school	6	10.34	
Other	1	1.72	
Relationship of caregiver to child			
Grandparent	36	64.29	
Great grandparent	4	7.14	
Aunt or uncle	4	7.14	
Great-aunt or -uncle	6	10.71	
Cousin	4	7.14	
Other	2	3.57	
Relationship of caregiver to birth parent (child’s mother or child’s father)			
Mother	40	72.73	
Father	15	27.27	
Do you have legal guardianship?			
Yes	26	45.61	
No	30	52.63	
Adopted or birth child	1	1.75	
Marital status of caregiver			
Married	17	29.31	
Divorced	7	12.07	
Separated	8	13.79	
Widowed	4	6.90	
Single	22	37.93	
Employment status of caregivers			
No	31	54.39	
Yes, part-time	5	8.77	
Yes, full-time	21	36.84	
Formal or informal care			
Formal	17	31.48	
Informal	37	68.52	

Note. [i] Valid percentages were calculated based on the number of valid responses for each variable.

**Table 2:** Depression.

Variable	β	*SE*	*p* Value	LL95	UL95	Std. β
Intercept	0.558	0.047	0.000	0.466	0.650	1.300
Child Age	0.013	0.028	0.637	−0.042	0.068	0.070
Family Resources	0.000	0.034	0.992	−0.067	0.067	0.001
Family Functioning	0.009	0.006	0.117	−0.003	0.021	0.232
Birth Mother–Child Relationship	−0.132	0.056	0.019	−0.242	−0.022	−0.238
Cultural Pride	0.131	0.090	0.147	−0.045	0.307	0.195
Alertness to Racism	−0.256	0.101	0.011	−0.454	−0.058	−0.283
Interracial Coping	−0.022	0.215	0.917	−0.443	0.399	−0.016
Caregiver Stress	0.310	0.089	0.001	0.136	0.484	0.341
Child Gender (female)	−0.021	0.132	0.874	−0.280	0.238	−0.023
Annual Income	−0.032	0.019	0.086	−0.069	0.005	−0.238
Informal Kinship Care	0.150	0.113	0.184	−0.071	0.371	0.162
Length of Stay	−0.007	0.009	0.465	−0.025	0.011	−0.100
Caregiver Age	−0.052	0.025	0.039	−0.101	−0.003	−0.260

**Table 3:** Anxiety.

Variable	β	*SE*	*p* Value	LL95	UL95	Std. β
Intercept	0.779	0.046	0.000	0.689	0.869	1.599
Child Age	−0.017	0.028	0.546	−0.072	0.038	−0.081
Family Resources	−0.046	0.041	0.256	−0.126	0.034	−0.165
Health Competency	0.009	0.007	0.200	−0.005	0.023	0.204
Birth Mother–Child Relationship	−0.025	0.053	0.631	−0.129	0.079	−0.04
Cultural Pride	0.241	0.104	0.021	0.037	0.445	0.315
Alertness to Racism	−0.378	0.163	0.021	−0.697	−0.059	−0.368
Interracial Coping	0.273	0.218	0.210	−0.154	0.700	0.171
Caregiver Stress	0.357	0.14	0.011	0.083	0.631	0.345
Child Gender (female)	−0.044	0.121	0.717	−0.281	0.193	−0.042
Annual Income	−0.032	0.022	0.135	−0.075	0.011	−0.212
Informal Kinship Care	−0.045	0.137	0.743	−0.314	0.224	−0.043
Length of Stay	0.011	0.008	0.168	−0.005	0.027	0.148
Caregiver Age	−0.034	0.026	0.184	−0.085	0.017	−0.152

## Data Availability

The datasets presented in this article are not readily available because of IRB policies concerning participant confidentiality and the data are part of an ongoing study.

## Supplementary Materials

The following supporting information can be downloaded at https://www.mdpi.com/article/10.3390/healthcare13162025/s1, Table S1: CARES Subscales “Cultural Legacy” and “Interracial Coping” with Depression. Table S2: CARES Subscales “Racial & Religious Coping” and “Interracial Coping” with Depression. Table S3: CARES Subscales “Cultural Pride” and “Promotion of Distrust” with Depression. Table S4: CARES Subscales “Cultural Legacy” and “Interracial Coping” with Anxiety. Table S5: CARES Subscales “Racial & Religious Coping” and “Interracial Coping” with Anxiety. Table S6: CARES Subscales “Cultural Pride” and “Promotion of Mistrust” with Anxiety.
