# Supplementary material for: Caregiver and Birth Parent Influences on Depression and Anxiety in African American Children in Kinship Care
Source: Healthcare (Basel). 2025 Aug 17;13(16):2025. doi: 10.3390/healthcare13162025 (PMC12385733; doi:10.3390/healthcare13162025)
Supplement: Supplementary file 1 [file healthcare-13-02025-s001.zip › healthcare-3729085-supplementary.pdf]

Appendix 1. Collinear CARES Subscales

Table S1: CARES Subscales “Cultural Legacy” and “Interracial Coping” with Depression.

**Variable**	**β**	***SE***	***p* Value**
Intercept	0.562	0.047	0.000
Child age	0.014	0.029	0.643
Family resources	0.008	0.037	0.819
Family functioning	0.008	0.006	0.128
Child relationship with birth mother	-0.142	0.060	0.018
Cultural Legacy	0.067	0.102	0.512
Alertness to racism	-0.199	0.102	0.050
Interracial Coping	-0.014	0.247	0.956
Caregiver stress	0.313	0.091	0.001
Child gender (female)	-0.014	0.136	0.918
Annual income	-0.035	0.020	0.087
Informal kinship care	0.176	0.123	0.152
Length of stay	-0.009	0.010	0.374
Caregiver age	-0.049	0.026	0.058

R2 = 0.486

Table S2: CARES Subscales “Racial & Religious Coping” and “Interracial Coping” with Depression.

**Variable**	**β**	***SE***	***p* Value**
Intercept	0.565	0.046	0.000
Child age	0.011	0.027	0.698
Family resources	0.005	0.036	0.879
Family functioning	0.008	0.005	0.158
Child relationship with birth mother	-0.134	0.057	0.019
Racial & Religious Coping	0.105	0.157	0.503
Alertness to racism	-0.221	0.118	0.062
Interracial Coping	0.013	0.215	0.950
Caregiver stress	0.323	0.089	0.000
Child gender (female)	-0.030	0.125	0.811
Annual income	-0.035	0.020	0.078
Informal kinship care	0.171	0.120	0.155
Length of stay	-0.008	0.009	0.337
Caregiver age	-0.047	0.025	0.058

R2 = 0.485

Table S3: CARES Subscales “Cultural Pride” and “Promotion of Distrust” with Depression.

Variable	β	*SE*	*p* Value
Intercept	0.561	0.046	0.000
Child age	0.011	0.027	0.684
Family resources	-0.003	0.031	0.912
Family functioning	0.009	0.006	0.110
Child relationship with birth mother	-0.130	0.055	0.018
Cultural pride	0.124	0.093	0.180
Alertness to racism	-0.268	0.100	0.008
Promotion of Mistrust	0.044	0.149	0.768
Caregiver stress	0.307	0.094	0.001
Child gender (female)	-0.033	0.128	0.799
Annual income	-0.030	0.019	0.115
Informal kinship care	0.146	0.110	0.186
Length of stay	-0.007	0.009	0.468
Caregiver age	-0.051	0.022	0.021

R2 = 0.506

Table S4: CARES Subscales “Cultural Legacy” and “Interracial Coping” with Anxiety.

Variable	β	*SE*	*p* Value
Intercept	0.785	0.046	0
Child age	-0.023	0.026	0.38
Family resources	-0.028	0.044	0.518
Family functioning	0.009	0.007	0.188
Child relationship with birth mother	-0.048	0.045	0.284
Cultural Legacy	0.216	0.115	0.059
Alertness to racism	-0.326	0.168	0.052
Interracial Coping	0.212	0.24	0.377
Caregiver stress	0.355	0.144	0.014
Child gender (female)	-0.018	0.135	0.896
Annual income	-0.038	0.023	0.1
Informal kinship care	-0.032	0.138	0.818
Length of stay	0.009	0.008	0.241
Caregiver age	-0.032	0.026	0.22

R2 = 0.515

Table S5: CARES Subscales "Racial & Religious Coping" and "Interracial Coping" with Anxiety.

Variable	β	*SE*	*p* Value
Intercept	0.789	0.049	0
Child age	-0.025	0.027	0.369
Family resources	-0.037	0.043	0.392
Family functioning	0.007	0.006	0.289
Child relationship with birth mother	-0.027	0.054	0.619
Racial & Religious Coping	0.213	0.169	0.209
Alertness to racism	-0.323	0.192	0.092
Interracial Coping	0.317	0.258	0.219
Caregiver stress	0.393	0.146	0.007
Child gender (female)	-0.061	0.126	0.627
Annual income	-0.038	0.024	0.121
Informal kinship care	-0.021	0.152	0.891
Length of stay	0.009	0.009	0.315
Caregiver age	-0.024	0.025	0.335

R2 = 0.495

Table S6: CARES Subscales "Cultural Pride" and "Promotion of Mistrust" with Anxiety.

Variable	β	*SE*	*p* Value
Intercept	0.769	0.046	0
Child age	-0.011	0.03	0.709
Family resources	-0.029	0.036	0.431
Family functioning	0.011	0.007	0.137
Child relationship with birth mother	-0.031	0.056	0.576
Cultural pride	0.255	0.098	0.009
Alertness to racism	-0.299	0.147	0.043
Promotion of Mistrust	-0.03	0.168	0.857
Caregiver stress	0.346	0.141	0.014
Child gender (female)	0.005	0.119	0.966
Annual income	-0.036	0.022	0.098
Informal kinship care	-0.026	0.13	0.843
Length of stay	0.011	0.008	0.167
Caregiver age	-0.04	0.025	0.112

R2 = 0.519
